# Bibliometric analysis of sugammadex sodium literature based on the Web of Science database

**DOI:** 10.1097/MD.0000000000046464

**Published:** 2026-05-12

**Authors:** Jinjiang Liu, Xiaowen Lei, Xu Wen, Jing Zhao, Yong Guo, Rui Cao

**Affiliations:** a Department of Anesthesiology, Mianyang 404 Hospital, Mianyang, China; b Department of Anesthesiology, Zitong County People’s Hospital, Zitong, China.

**Keywords:** bibliometric analysis, bibliometrix R, CiteSpace, sugammadex sodium, Web of Science

## Abstract

**Objective:**

This study employed bibliometric techniques to conduct both qualitative and quantitative analyses of literature pertaining to sugammadex sodium. The aim was to uncover the trends and hotspots of research in this field.

**Methods:**

Publications on sugammadex sodium published from January 2002 to December 2024 were extracted from the Web of Science Core Collection. Statistical analysis and visualization of publication data were performed using CiteSpace, Bibliometrix, and Excel.

**Results:**

A total of 1006 articles were analyzed, including 3922 researchers from 56 countries/regions and 933 institutions. These studies have been published in 305 journals. Although Dutch scholars have demonstrated greater influence, the United States boasted the highest output. Among these institutions, Merck Germany had the highest publication volume and impact. Dutch researcher De Boer HD ranked the highest in terms of publication volume and influence. The British Journal of Anesthesia was the leading journal in terms of publications on sugammadex sodium, with a total of 59 articles. The most cited article in the field was by Kheterpal and colleagues, published in Anesthesiology in 2020. Keyword analysis revealed frequent comparisons of sugammadex sodium and neostigmine. Research hotspots have primarily focused on the reversal of rocuronium-induced neuromuscular blockade, deep neuromuscular blockade reversal, impact on residual neuromuscular blockade, and postoperative complications. The effects of sugammadex sodium on postoperative complications, particularly pulmonary complications, represent a current research frontier.

**Conclusion:**

Research on sugammadex sodium has received significant scholarly attention, with strong potential for future collaborative studies. The impact of sugammadex sodium on postoperative complications remains a promising area for in-depth analysis and a topic for future research.

## 1. Introduction

Sugammadex sodium is the first selective antagonist of aminosteroid neuromuscular blocking agents. Its molecular structure features an 11 Å hollow hydrophobic conical cavity composed of 8 cyclic oligosaccharides and peripheral side chains.^[1,2]^ This configuration facilitates binding with amino steroid neuromuscular blocking agents, thereby diminishing their concentration in the bloodstream and promoting the movement of these agents from the neuromuscular junction back into the blood, ultimately reversing the effects of neuromuscular blockade.^[3]^ Sugammadex sodium is characterized by its rapid onset of action, minimal side effects, and broad applicability in antagonizing amino steroid neuromuscular blocking agents.^[4–6]^ Compared with traditional muscle relaxant antagonists such as neostigmine, sugammadex sodium offers several advantages, including independence from blockade depth, reduced risk of airway complications, and lack of interference with the cholinergic system.^[7–11]^ Consequently, the American Society of Anesthesiologists recommends it as the preferred antagonist for amino steroid neuromuscular blocking agents. Sugammadex sodium was first reported for clinical use in 2005^[12]^ and has since been approved in various regions, including the United States and China. With the expiration of its patent and the subsequent introduction of generic versions, a significant reduction in the cost of the drug is anticipated, which is likely to spur a trend of increasing clinical applications and research into sugammadex sodium.

Despite the extensive body of literature on sugammadex sodium, there remains a lack of comprehensive and systematic bibliometric analyses on this subject. Bibliometrics is an academic discipline that employs quantitative and qualitative methods to statistically analyze information from publications, assess research trends, identify hotspots, and understand contributions across different countries, institutions, and authors.^[13]^ This study aimed to apply bibliometric techniques to systematically analyze the global scientific output of sugammadex sodium, revealing its developmental trends within the field of anesthesiology and providing references for future related research.

## 2. Materials and methods

### 2.1. Data collection

**Figure 1.**
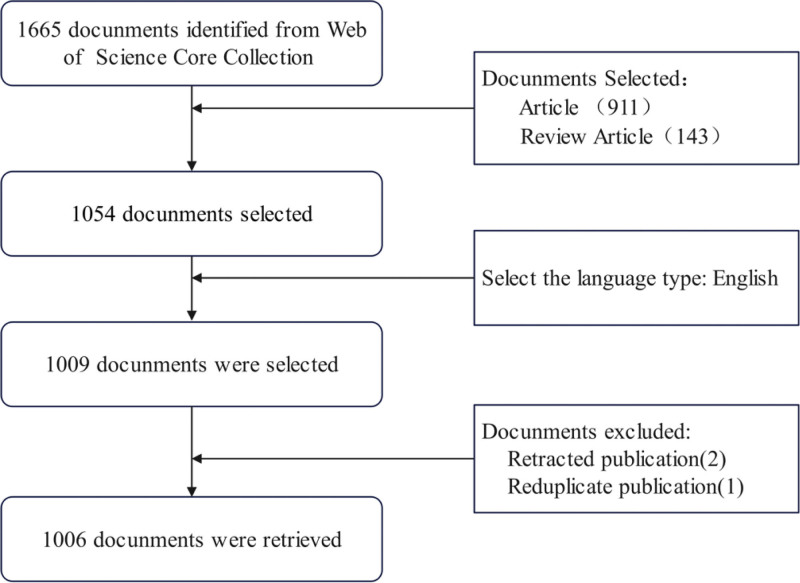
Search flowchart.

The data utilized in this study were sourced exclusively from the Web of Science Core Collection, specifically the Science Citation Index Expanded and the Emerging Sources Citation Index subdatabases. Relevant publications before 2002 were nonexistent; thus, the retrieval was confined to the period between January 1, 2002, and December 30, 2024. Before searching, medical subject headings were used to identify and refine the synonyms for sugammadex sodium, which were then incorporated into our search strategy. The search query was formulated as follows: TI = (“Sugammadex sodium” OR “Org 25969” OR “Sugammadex sodium Sodium” OR “Bridion”) OR AB = (“Sugammadex sodium” OR “Org 25969” OR “Sugammadex sodium Sodium” OR “Bridion”) OR AK = (“Sugammadex sodium” OR “Org 25969” OR “Sugammadex sodium Sodium” OR “Bridion”). Furthermore, the search adhered to specific exclusion criteria, eliminating document types such as “letter,” “meeting abstract,” “editorial material,” “correction,” and “news item” to mitigate bias. Only “article” and “review article” types were included in the analysis, and the language was restricted to English. The results were meticulously reviewed by 2 independent researchers to eliminate duplicate records and irrelevant literature. Relevant data were downloaded from the Web of Science Core Collection on January 10, 2025. The search flowchart is shown in Figure 1. Since this study is a bibliometric analysis based exclusively on data from a public database and does not involve any direct contact with human or animal subjects, ethical approval was not required for this work.

### 2.2. Data analysis

For bibliometric analysis, several software tools were employed, including CiteSpace (version 6.3.R1; Drexel University, Philadelphia), Bibliometrix (R package version 4.4.0; University of Naples “Parthenope”, Naples, Italy), and Microsoft Excel (2019; Microsoft Corporation, Redmond).

CiteSpace, an open-source software developed by Professor Chaomei Chen on the JAVA platform, was designed for the analysis and visualization of scientific literature, focusing on revealing the structure of knowledge and its dynamic evolution within academic research.^[14]^ The visual maps generated by this software encompass various bibliometric indicators such as nations, institutions, authors, journals, and cited references. Larger nodes represent higher frequencies of occurrence, whereas the lines between nodes denote specific relationships, such as collaboration, co-citation, and co-occurrence, aimed at uncovering connections within academic research. Nodes surrounded by a purple halo indicate betweenness centrality >0.1, with a more pronounced halo signifying greater importance within the knowledge network. In keyword clustering analysis, a modularity value (*Q*) >0.3 and an average silhouette score (*S*) >0.5 indicate high-quality clustering results.^[15]^ Moreover, Bibliometrix software was utilized for the visualization analysis of core journals and authors, while Excel was used for the visualization of annual publication volume, countries, and institutions.

## 3. Results

A total of 1006 articles were included in the analysis, of which 865 were research articles (86%) and 141 were review articles (accounting for the remaining 14%). As of December 30, 2024, studies involving sugammadex sodium had accrued 19,470 citations. After excluding self-citations, the net citation count was 10,769, with an average citation rate of 19.3 per article and an H-index of 68.

### 3.1. Annual publication analysis

**Figure 2.**
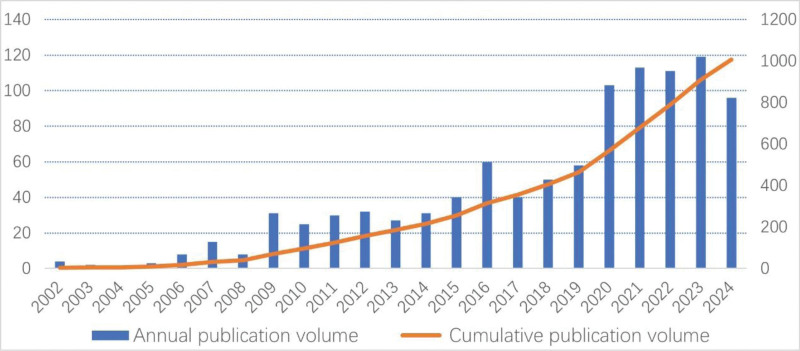
Analysis of publication volume for sugammadex sodium research from 2002 to 2024.

From January 1, 2022, to December 30, 2024, the total number of publications related to sugammadex sodium reached 1006, averaging 42 publications per year. The annual publication volume, along with cumulative publication trends, is shown in Figure 2. The blue bars in the graph represent annual publication volume. Between 2002 and 2008, the annual output of this field was consistently below 20. Since 2008, there has been a remarkable increase compared to the initial phase of research, with the cumulative publication volume (represented by the red solid line in the graph) demonstrating a continuous and stable upward trajectory. Notably, the period from 2017 to 2021 saw the highest growth rate in annual publications, with the cumulative volumes exhibiting exponential growth.

### 3.2. National/regional analysis

**Figure 3.**
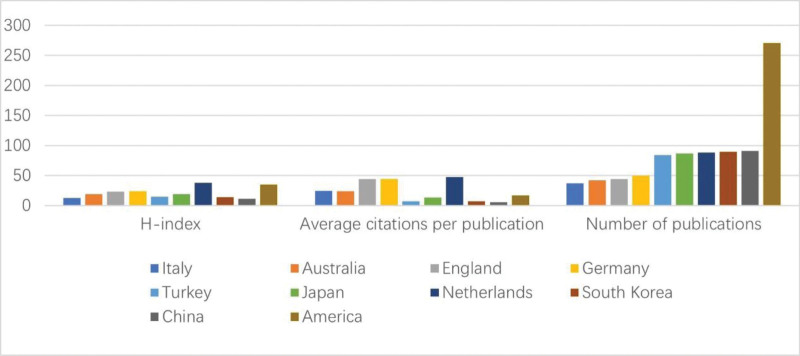
Analysis of the top 10 countries/regions by publication volume for sugammadex sodium.

**Figure 4.**
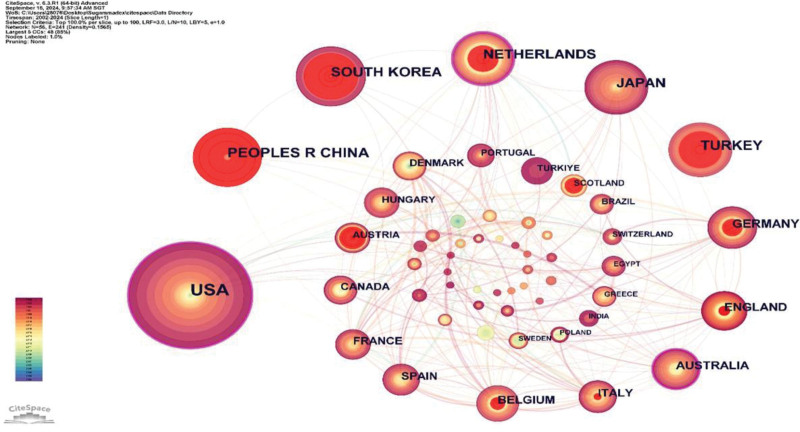
Network map of national/regional collaborations in sugammadex sodium research.

Researchers from 56 countries and regions have published articles related to sugammadex sodium. An analysis of the top 10 countries by publication volume is presented in Figure 3, with the United States (271 articles), China (91 articles), and South Korea (90 articles) leading in publication output. In terms of article impact, the H-index and average citation count per article are critical indicators of scholarly contribution.^[16]^ Dutch publications are of high quality and have wide-reaching impacts, receiving extensive recognition and citation within the academic community. By contrast, articles from South Korea and China, which started their research later, had a comparatively lower impact. The visualization of national co-occurrence in Figure 4 highlights the Netherlands (0.21), the United States (0.21), and Australia (0.13) as having high betweenness centrality, indicating that these countries play a pivotal role as bridges in scientific collaboration, linking various other nations. The connections between nodes reveal close cooperation between the United States and countries such as the Netherlands, Australia, and the United Kingdom. The nodes representing the United States transition from central yellow to peripheral red, illustrating a consistent and stable output in sugammadex sodium research by American scholars. Conversely, the nodes for China and South Korea, primarily shown in red or orange, combined with their annual publication volumes, suggest that these countries started their research later but have recently become active in the field.

### 3.3. Institutional analysis

**Figure 5.**
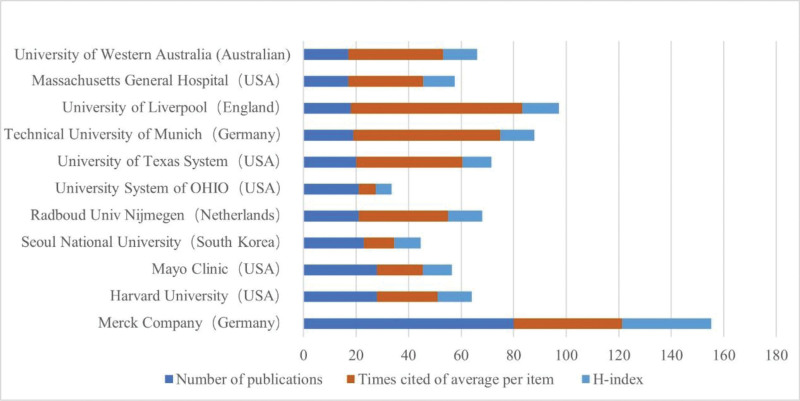
Analysis of the output of the top 10 institutions by publication volume.

**Figure 6.**
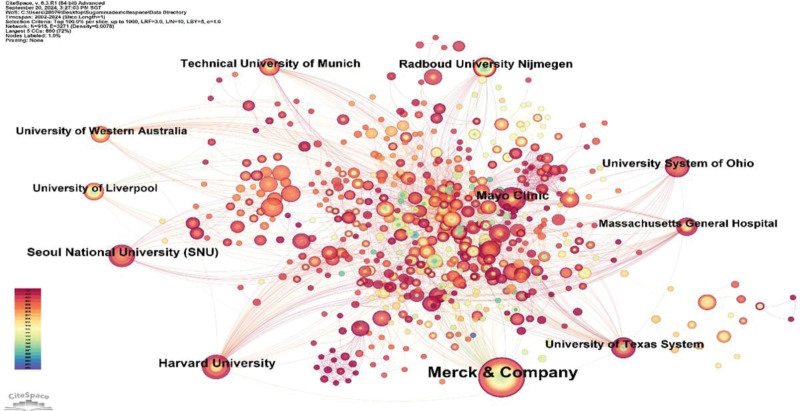
Network diagram of institutional collaborations in sugammadex sodium research.

Globally, researchers from 933 institutions have published papers in this field. An analysis of the top 10 institutions by publication volume is illustrated in Figure 5, with Merck in Germany leading significantly with 80 publications, far surpassing the second-place tie between Harvard University and Mayo Clinic, both with 28 publications. Moreover, Merck’s H-index was 34, which is distinctly higher than that of any other institution. However, in terms of the average citation rate per article, the University of Liverpool in the United Kingdom ranks first, with an average of 64.89 citations per publication. Figure 6 displays a network diagram of institutional collaborations, highlighting extensive collaborations between the top 10 publishing institutions and other research entities, although direct collaborations among the top 10 are relatively limited.

### 3.4. Author analysis

**Figure 7.**
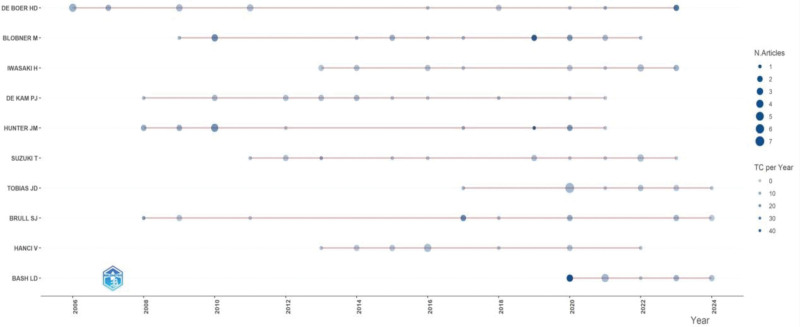
Temporal changes in the output of the top 10 authors in sugammadex sodium research.

**Figure 8.**
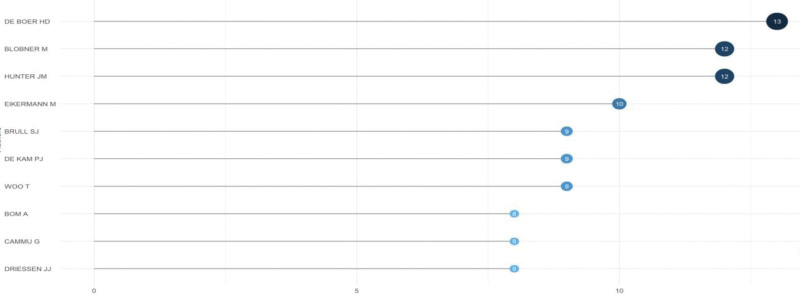
H-index of the top 10 authors in the sugammadex sodium research field.

A total of 3992 researchers globally have contributed to the research on sugammadex sodium. The top 10 authors by publication volume are depicted in Figure 7, with De Boer HD from the Netherlands demonstrating a consistent and stable output in the sugammadex sodium research field, having published 19 articles. The H-index analysis, as shown in Figure 8, indicated that De Boer HD was the most influential author in this domain, with an H-index of 13.

Price’s law suggests that a stable core group of authors is formed when the number of papers published by core authors in a domain reaches or exceeds 50% of the total number of publications in that field.^[17]^ The formula used for calculation was *M* = 0.749(Nmax)^(1/2)^, where Nmax represents the highest publication count (Nmax = 19), resulting in *M* = 4. Thus, in the field of sugammadex sodium research, authors who have published at least 4 papers are considered the core authors. Statistics show that 144 authors published 4 or more papers, with a combined total of 375 publications, accounting for 38% of the sample literature. This indicates that the sugammadex sodium research field has not yet established a stable core group of high-output authors, and the number of core authors needs to be further increased.

### 3.5. Journal analysis

A total of 305 journals have published articles on sugammadex sodium. The top 10 journals with the highest publication volume are listed in Table 1, collectively contributing to 326 articles. Among these, 60% were classified within the first quartile of the Journal Citation Reports, 30% in the second quartile, and 10% in the third quartile. The British Journal of Anesthesia had the highest number of publications on sugammadex sodium, totaling 59 articles, followed by Anesthesia and Analgesia with 46 articles, and BMC Anesthesiology with 37 articles. Among the top 10 journals, articles published in Anesthesiology had the highest average citation rate of 92, with the British Journal of Anaesthesia and Anesthesia, followed by 53 and 46 average citations, respectively.

**Table 1:** The top 10 journals publishing articles related to sugammadex sodium.

Journal name	Number of publications	JCR category	Impact factor	Average citations per article	H-index
*British Journal of Anaesthesia*	59	Q1	9.1	53.95	36
*Anesthesia and Analgesia*	46	Q1	4.6	38.13	20
*BMC Anesthesiology*	37	Q2	2.3	12	13
*Anesthesiology*	31	Q1	9.1	92.48	26
*Anaesthesia*	29	Q1	7.5	44.62	22
*Journal of Anesthesia*	29	Q2	2.8	12.59	12
*Journal of Clinical Anesthesia*	26	Q1	5	23.08	17
*Current Opinion in Anesthesiology*	24	Q3	2.3	14.42	13
*European Journal of Anaesthesiology*	23	Q1	4.2	26.13	11
*Medicine*	22	Q2	1.3	6.45	7

JCR = Journal Citation Reports.

### 3.6. Analysis of references

**Figure 9.**
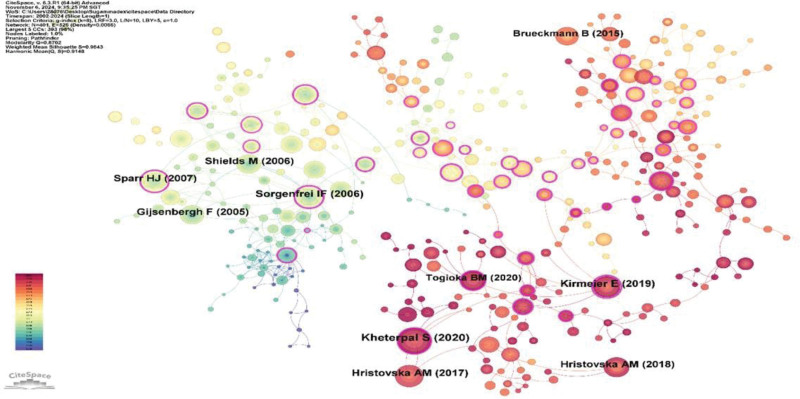
Co-occurrence analysis of references on sugammadex sodium.

The examination of references not only facilitates the rapid identification of pivotal citations relevant to sugammadex sodium research by scholars in the field but also indirectly reflects the research direction.^[18]^ The most cited article in the field of sugammadex sodium research was by Kheterpal et al^[19]^ published in 2020 in the journal Anesthesiology, while the publication with the highest impact factor was by Kirmeier et al^[20]^ published in 2019 in Lancet Respiratory Medicine. The co-occurrence analysis of references, as shown in Figure 9, illustrates the citation frequency by node size and the co-citation relationships by connecting lines, with different colors representing different time periods.

### 3.7. Emergence analysis of references

**Figure 10.**
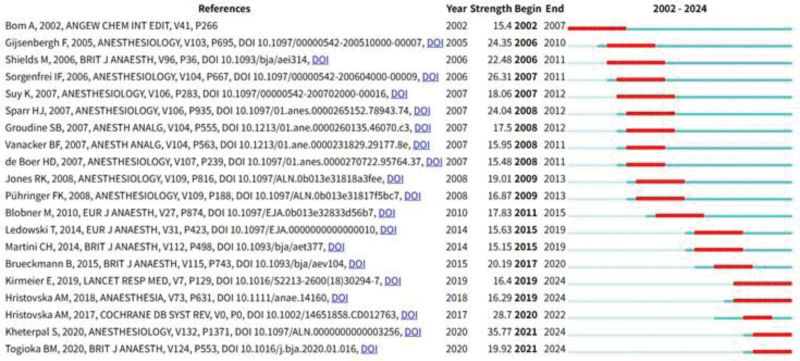
Emergence analysis of references on sugammadex sodium.

This analysis reveals references that have experienced a sharp increase in citation frequency within specific timeframes, often correlating with research hotspots. Figure 10 displays the top 20 emerging references, 4 of which continue to exert a significant influence on current sugammadex sodium research.

**Figure 11.**
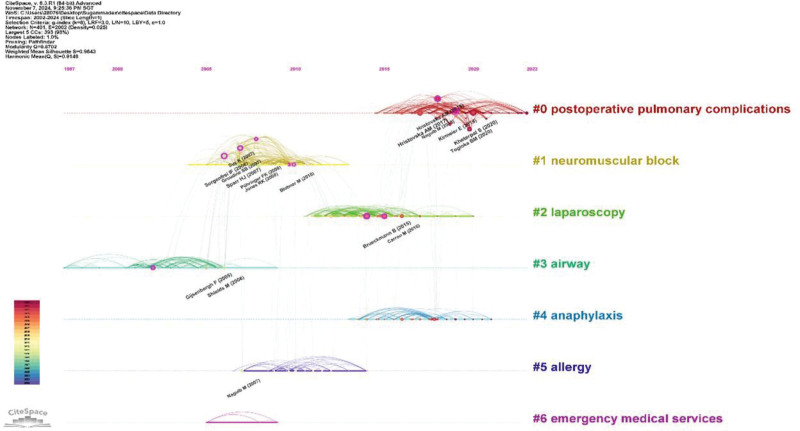
Clustered timeline of references on sugammadex sodium.

Figure 11 presents a clustered timeline of the references for sugammadex sodium. This timeline graph clusters referenced articles cited by similar research themes, as indicated by the keywords from the cited articles. Nodes represent cited articles, and nodes on the same horizontal line belong to the same cluster. The timeline extends to the present, showing ongoing relevance, particularly for cluster “0# Postoperative pulmonary complications.”

### 3.8. Keyword analysis

**Figure 12.**
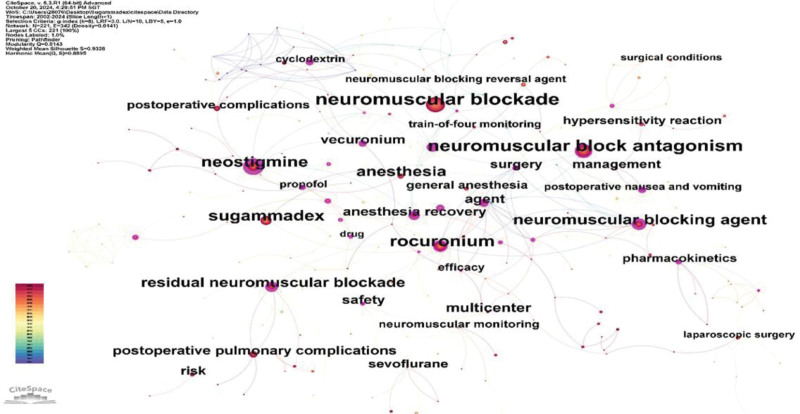
Co-occurrence analysis graph of keywords in sugammadex sodium literature.

Figure 12 illustrates the co-occurrence analysis of the keywords, where the font size of the node labels represents their frequency of occurrence. A larger font size indicates higher frequency. The 10 most frequent keywords and their occurrences are as follows: “neuromuscular blockade” (459 occurrences), “neuromuscular block antagonism” (339 occurrences), “rocuronium” (257 occurrences), “Sugammadex sodium” (224 occurrences), “neostigmine” (223 occurrences), “neuromuscular blocking agent” (182 occurrences), “anesthesia” (157 occurrences), “residual neuromuscular blockade” (145 occurrences), “multicenter” (104 occurrences), and “vecuronium” vecuronium’(88 occurrences). The keywords “neuromuscular blockade,” “neuromuscular block antagonism,” “neuromuscular blocking agent,” and “anesthesia” reflect the essential pharmacological actions of sugammadex sodium in the field of anesthesia, specifically, the reversal of neuromuscular blockade, which forms the basis of research on sugammadex sodium. The keywords “Sugammadex sodium” and “neostigmine” exhibit similar frequencies and are tightly linked, while “rocuronium” appeared more frequently than “vecuronium.” The keyword “residual neuromuscular blockade” represents the specific research direction of sugammadex sodium, focusing on the effects of residual neuromuscular blockade.

**Figure 13.**
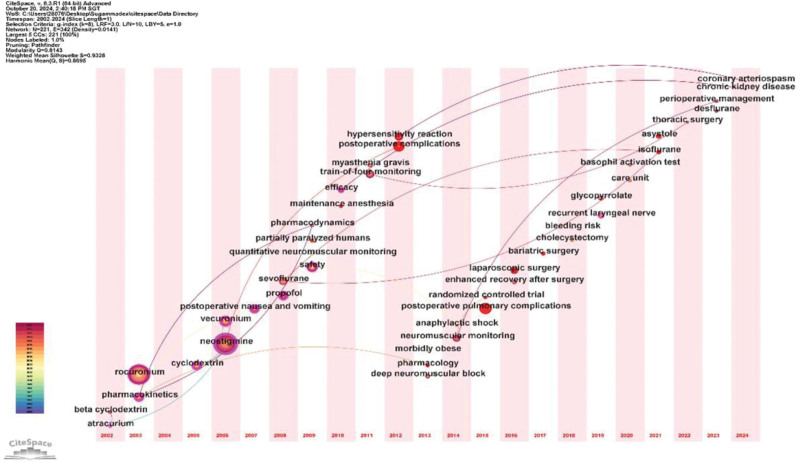
Timeline graph of keywords for sugammadex sodium.

In the timeline analysis of keywords, as depicted in Figure 13, we selected 2 to 3 keywords with the highest or most significant frequencies each year. The years listed below the keywords denote their first appearance in sugammadex sodium.^[16]^ Early research keywords included “beta cyclodextrin” (2002), “pharmacokinetics” (2003), “cyclodextrin” (2005), “sevoflurane” (2008), “pharmacodynamics” (2009), and “safety” (2009). Later keywords emerged, including “partially paralyzed humans” (2009), “postoperative complications” (2012), “hypersensitivity reaction” (2012), “morbidly obese” (2013), “postoperative pulmonary complications” (2015), “laparoscopic surgery” (2016), “bariatric surgery” (2017), “bleeding risk” (2018), “basophil activation test” (2020), “asystole” (2021), and “chronic kidney disease” (2024). This timeline reveals a shift from the initial focus on drug structure and pharmacology towards clinical research.

**Figure 14.**
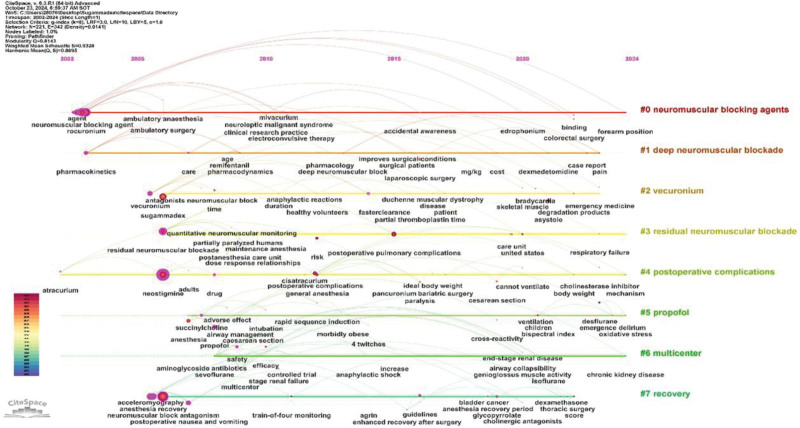
Sugammadex sodium keyword clustering timeline.

The keyword clustering timeline not only encompasses the clustering of keywords but also illustrates the temporal span of these keywords within the cluster. As depicted in Figure 14, clusters “#0” and “#2” show similarities to the results of the emergent keyword analysis, with sugammadex sodium’s antagonistic effects on neuromuscular blockade serving as the foundational research in this field. Furthermore, rocuronium antagonism by sugammadex sodium represents a principal research focus in this domain. Cluster “#5” signifies the application of sugammadex sodium to propofol anesthesia. Cluster “#6” includes keywords such as safety, anaphylactic shock, and chronic kidney disease, indicating that multicentric clinical studies have been conducted in these research directions with sugammadex sodium. Clusters “#1,” “#3,” “#4,” and “#7” represent specific research directions within the field of sugammadex sodium, highlighting the research focus areas concerning its effects on deep neuromuscular blockade, residual neuromuscular blockade, postoperative complications, and recovery.

**Figure 15.**
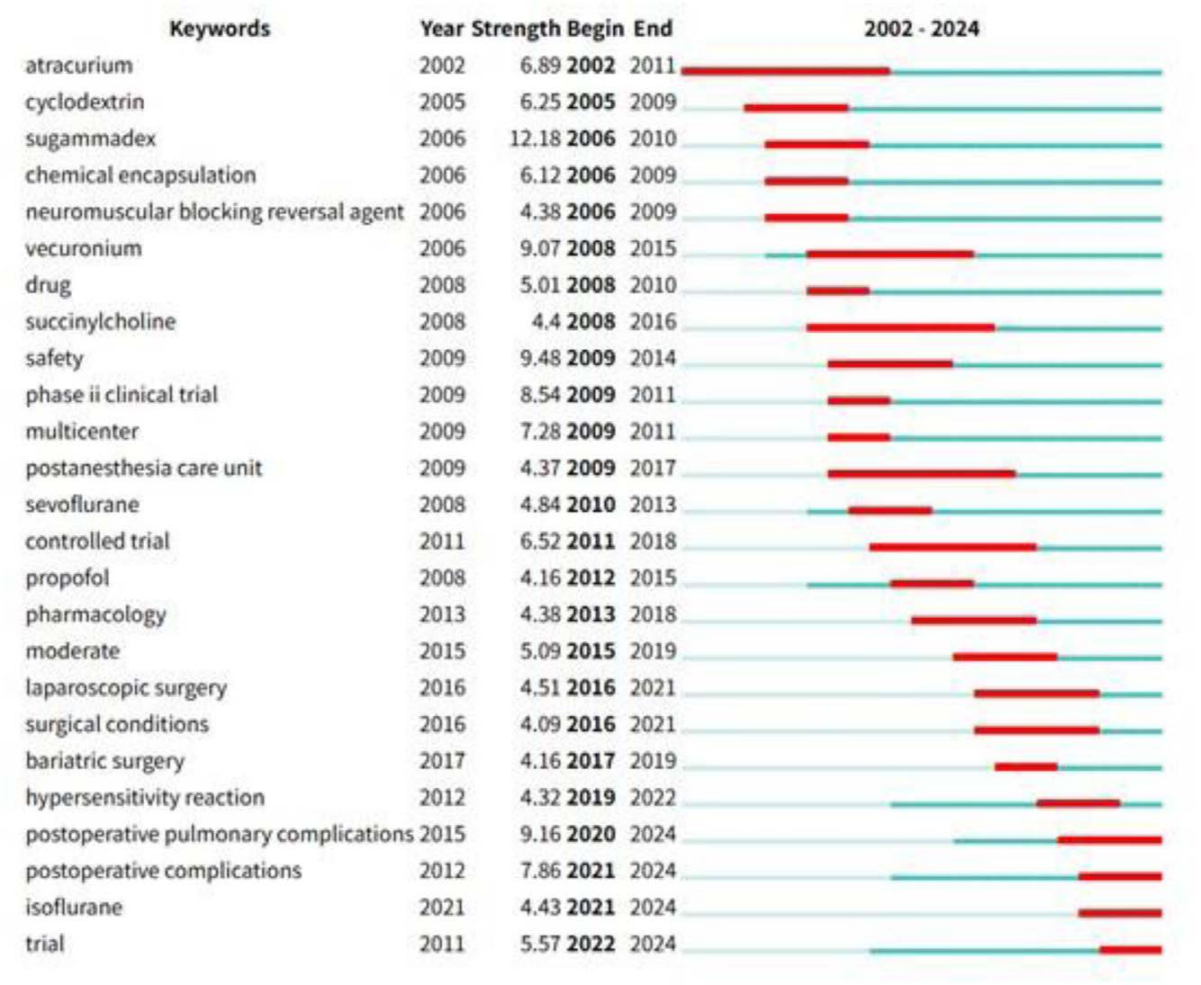
Sugammadex sodium emergent keyword analysis.

The analysis of emergent keywords, as shown in Figure 15, features keywords such as “postoperative pulmonary complications” and “postoperative complications,” which have continued to be prominent and are closely associated with research directions involving sugammadex sodium.

## 4. Discussion

Reviewing the publication volume over the past 2 decades revealed an overall upward trend in research related to sugammadex sodium. From 2002 to 2008, the field was in its nascent stages, with an annual publication volume consistently below 20 articles. In 2008, sugammadex sodium was first approved for use in adults in Europe and Australia, signifying a rapid development phase in related research with a substantial increase in publication volume compared to the initial stages. Following approval from various regions, such as the United States Food and Drug Administration in 2015 and the National Medical Products Administration of China in 2019, the period from 2017 to 2021 saw the greatest growth rate in annual publication volume, demonstrating a stable upward trend. The increase in publication volume reflects not only a global surge in scientific interest in sugammadex sodium but also its gradual clinical adoption worldwide. Despite the numerous advantages of sugammadex sodium in antagonizing aminosteroid neuromuscular blocking agents, the progression of related research has broadly coincided with its approval timelines in different regions, which is likely linked to its high usage costs. However, as patent protection for sugammadex sodium expires and various generic versions have been introduced to the market, the research field is expected to experience a new surge in growth.

The US holds a preeminent position in the domain of sugammadex sodium research, as evidenced by both the quantity and quality of its publications. This prominence is likely linked to early market approval and robust regional economic development. Although China later commenced its investigations into sugammadex sodium, its publication output ranks second only to the United States. Chinese scholars must intensify their research on sugammadex sodium to augment their influence in this field. Among the top 10 institutions by publication volume, half are located in the United States, aligning with the high publication output observed there. Merck & Co., as the principal manufacturer and marketer of sugammadex sodium, leads in both the volume of publications and the H-index among all institutions, indicating significant research investment and production of high-quality articles. Co-authorship network maps reveal collaborations between countries and institutions, although such collaborations are less frequent among high-output countries and institutions. To fully leverage the synergistic effects and enhance the value and impact of research on sugammadex sodium, it is crucial to strengthen collaboration between these high-output entities.

Among all journals, the British Journal of Anesthesia, a leading journal in the field of anesthesiology, has published the most articles on sugammadex sodium. Approximately 30% of the articles related to sugammadex sodium were published in journals ranked in the second quartile of Journal Citation Reports or higher. This not only indicates a significant production of high-quality articles but also reflects the favorable reception of this research in prestigious journals. Among all the authors, De Boer HD from the Netherlands has demonstrated a consistent and enduring contribution to sugammadex sodium research, while Lori Bash from the United States exhibits a rapidly increasing research output in this area. Despite the presence of such prolific authors, the field of sugammadex sodium research has yet to develop a core group of high-output researchers. This suggests a need for enhanced collaboration and consolidation of more researchers to improve the overall research quality and impact in this field.

The most frequently cited article is the study by Kheterpal et al,^[19]^ which primarily investigated the relationship between the use of antagonistic drugs for neuromuscular blockade and the incidence of postoperative pulmonary complications. The study with the highest impact factor was by Kirmeier et al,^[20]^ who focused on the effects of neuromuscular blocking agents on postoperative pulmonary complications. In the analysis of citation bursts, 4 articles have shown ongoing citation bursts. In addition to the aforementioned studies, the other 2 articles exhibiting sustained bursts are the research by Brueckmann et al,^[21]^ which explored the impact of sugammadex on the rate of residual neuromuscular blockade postsurgery, and the study by Togioka et al,^[22]^ which discusses the effects of sugammadex sodium and neostigmine in reversing neuromuscular blockade on the incidence of pulmonary complications in elderly patients undergoing prolonged surgeries. Citation burst analysis revealed that current research on postoperative pulmonary complications is heavily cited in recent articles related to sugammadex sodium. This finding aligns with the results of the reference clustering analysis. We hypothesize that the influence of sugammadex sodium on postoperative pulmonary complications represents a research frontier in this field, which is consistent with the keyword-related analysis outcomes of this study.

The analysis of key terms revealed that during the initial stages of research on sugammadex sodium, the primary focus was on the structure, pharmacokinetics, pharmacodynamics, drug interactions, and safety of the drug. As the clinical application of sugammadex sodium has gradually expanded, new research directions have emerged, including its pharmacological properties, applications in specific patient populations and surgical settings, and impact on postoperative complications. Through keyword co-occurrence analysis, it is evident that the reversal of rocuronium-induced neuromuscular blockade by sugammadex sodium has garnered significant scholarly interest, particularly compared to vecuronium. Comparative studies of neostigmine and traditional antagonists of neuromuscular blockade are common in this field. The effects of sugammadex sodium on reversing rocuronium-induced neuromuscular blockade, reversal of deep neuromuscular blockade, its impact on residual neuromuscular blockade, and its influence on postoperative complications constitute current research hotspots in this area. Specifically, the effect of sugammadex sodium on postoperative complications represents a cutting-edge research topic. The impact of sugammadex sodium on postoperative complications encompasses a broad research area, with postoperative pulmonary complications emerging as a significant subdiscipline. Moreover, a few preliminary studies have explored the impact of sugammadex sodium on other postoperative complications, such as emergence agitation, postoperative gastrointestinal function, urinary retention, and nausea and vomiting.^[23–26]^ However, these studies are typically characterized by small sample sizes, single-center designs, and a lack of multidimensional exploration, such as varying surgical types and patient demographics. Future research in this area is expected to delve more deeply into these aspects.

This study had several limitations. First, data were sourced from the Web of Science database. Although this database has broad coverage, it does not encompass all literature pertaining to sugammadex sodium, which could introduce certain biases into the results. Second, the field of sugammadex sodium research is evolving rapidly. Owing to the timeliness of the study, it does not include the most recent findings, particularly those published after the search period; thus, the results may exhibit some degree of latency.

In summary, research on sugammadex sodium has received considerable attention from researchers. Initially, studies were focused on the basic pharmacological characteristics and safety profiles of the drug. Over time, the focus has shifted towards the properties of the drug, its application in specific patient groups and surgical contexts, and its impact on postoperative complications. Reversal of rocuronium-induced neuromuscular blockade, reversal of deep neuromuscular blockade, effects on residual neuromuscular blockade, and impact on postoperative complications are current hotspots in this research area. Notably, the effects of sugammadex sodium on postoperative complications, particularly pulmonary complications, represent the cutting-edge of research in this field. Furthermore, future studies will need to deepen collaboration between nations and institutions.

## Author contributions

**Data curation**: Jinjiang Liu.

**Methodology**: Jinjiang Liu, Yong Guo, Rui Cao.

**Resources**: Jinjiang Liu.

**Software**: Jinjiang Liu, Jing Zhao, Rui Cao.

**Writing – original draft**: Jinjiang Liu, Xiaowen Lei, Xu Wen, Jing Zhao, Yong Guo, Rui Cao.

**Writing – review & editing**: Jinjiang Liu, Xiaowen Lei, Xu Wen, Jing Zhao, Yong Guo, Rui Cao.

**Formal analysis**: Xiaowen Lei, Xu Wen, Rui Cao.

**Visualization**: Xiaowen Lei, Rui Cao.

**Funding acquisition**: Yong Guo.
